# Dihydro-sphingosine 1-phosphate interacts with carrier proteins in a manner distinct from that of sphingosine 1-phosphate

**DOI:** 10.1042/BSR20181288

**Published:** 2018-10-31

**Authors:** Yuko Mishima, Makoto Kurano, Tamaki Kobayashi, Masako Nishikawa, Ryunosuke Ohkawa, Minoru Tozuka, Yutaka Yatomi

**Affiliations:** 1 Department of Clinical Laboratory Medicine, The University of Tokyo, Tokyo, Japan; 2 Analytical Laboratory Chemistry, Graduate School of Health Care Sciences, Tokyo Medical and Dental University (TMDU), Tokyo, Japan; 3 Department of Immunology, Faculty of Health Sciences Kyorin University, Tokyo, Japan; 4 Analytical Laboratory Chemistry, Graduate School of Medical and Dental Sciences, Tokyo Medical and Dental University (TMDU), Tokyo, Japan

**Keywords:** apolipoprotein M, dihydro-sphingosine 1-phosphate, HDL, sphingosine 1-phosphate

## Abstract

Dihydro-sphingosine 1-phosphate (DH-S1P) is an analog of sphingosine 1-phosphate (S1P), which is a potent lysophospholipid mediator. DH-S1P has been proposed to exert physiological properties similar to S1P. Although S1P is known to be carried on HDL via apolipoprotein M (apoM), the association between DH-S1P and HDL/apoM has not been fully elucidated. Therefore, in the present study, we aimed to elucidate this association and to compare it with that of S1P and HDL/apoM. First, we investigated the distributions of S1P and DH-S1P among lipoproteins and lipoprotein-depleted fractions in human serum and plasma samples and observed that both S1P and DH-S1P were detected on HDL; furthermore, elevated amounts of DH-S1P in serum samples were distributed to the lipoprotein-depleted fraction to a greater degree than to the HDL fraction. Concordantly, a preference for HDL over albumin was only observed for S1P, and not for DH-S1P, when the molecules were secreted from platelets. Regarding the association with HDL, although both S1P and DH-S1P prefer to bind to HDL, HDL preferentially accepts S1P over DH-S1P. For the association with apoM, S1P was not detected on HDL obtained from apoM knockout mice, while DH-S1P was detected. Moreover, apoM retarded the degradation of S1P, but not of DH-S1P. These results suggest that S1P binds to HDL via apoM, while DH-S1P binds to HDL in a non-specific manner. Thus, DH-S1P is not a mere analog of S1P and might possess unique clinical significance.

## Introduction

Sphingosine-1-phosphate (S1P) is a bioactive lysophospholipid mediator that exerts various physiological functions through S1P receptors located on cell surfaces, such as antiapoptosis [1], cell proliferation [2], vasorelaxation, and the maintenance of vascular permeability [3–5]. Although the main source of plasma S1P, which has a concentration of ∼400 nmol/l in men and ∼350 nmol/l in women, is erythrocytes [6,7], S1P can also be derived from activated platelets [8] and endothelial cells [9]. Regarding the dynamism of S1P in circulation, apolipoprotein M (apoM) has crucial roles; the distribution of plasma S1P is unique compared with that of other lysophospholipids, with about two-thirds of S1P carried on HDL and the other one-third carried on albumin [10]. In 2011, Christoffersen et al. [11] reported that apoM is a carrier for S1P on HDL. Moreover, apoM reportedly modulates S1P metabolism: apoM can retard the degradation of S1P by lipid phosphate phosphatase or S1P lyase [12].

Although S1P is a representative sphingo-lysophospholipid mediator, dihydro-sphingosine 1-phosphate (DH-S1P), an analog of S1P, is another potent sphingo-lysophospholipid mediator. DH-S1P lacks one double bond in the 4-5 carbon position of S1P (Supplementary Figure S1), and its concentration in human plasma is 20–30% of that of S1P [6]. DH-S1P has been reported to activate S1P receptors (S1P1, S1P3, S1P4, and S1P5) and has been deemed to possess potent bioactivities similar to S1P [13]. However, although S1P possesses intracellular biological roles as well as agonist properties toward S1P receptors [14], DH-S1P was reported not to function intracellularly [15]. Regarding the metabolism of S1P and DH-S1P, their biogenesis pathways are slightly different: dihydro-sphingosine (DH-Sph) is generated by the serine and palmitoyl CoA and is subsequently phosphorylated by sphingosine kinase to DH-S1P, while sphingosine (Sph) is generated by ceramide and is subsequently phosphorylated by sphingosine kinase to produce S1P [16]. Moreover, although apoM has been elucidated to be an important molecule determining the concentration and dynamism of S1P, the association between apoM/HDL and DH-S1P remains to be elucidated.

With this background in mind, we sought to determine the distribution and protein binding of DH-S1P, compared with those of S1P, especially regarding the association with apoM/HDL.

## Materials and methods

### Separation and preparation of lipoproteins

Blood samples were collected from healthy human volunteers who had provided written informed consent, and acid citrate dextrose solution was immediately added as an anticoagulant. We then separated the lipoprotein fraction using ultracentrifugation. The sample was fractionated according to the following density ranges: 1.019–1.063 for LDL, 1.063–1.21 for HDL, and >1.21 for the lipoprotein**-**depleted (Lp-dep) fraction. In some experiments, we further fractionated the HDL as follow: 1.063–1.125 for HDL2, 1.125–1.21 for HDL3, and 1.21–1.25 for VHDL. HDL was dialyzed against PBS for 48 h before being used in the *in vitro* experiments. The present study was approved by the Institutional Research Ethics Committee of the Faculty of Medicine, The University of Tokyo (11158).

### Investigation of C_17_S1P and C_17_DH-S1P secretion from platelets or red blood cells

Washed platelets (50 × 10^4^/μl) were incubated with 10 μM C_17_Sph or C_17_DH-Sph for 5 min. After washing the platelets, 10 mg/ml of fatty acid-free albumin or HDL solution with 1 U/ml of thrombin was added to the washed platelets and stirred for 15 min. The samples were divided into supernatants and pellets by centrifugation, and the C_17_S1P and C_17_DH-S1P levels in these samples were measured.

The washed erythrocytes (100 × 10^4^/μl) were mixed with 10 mg/ml of albumin or HDL solution and incubated with 10 μM C_17_Sph or C_17_DH-Sph for 5 min. Then, the C_17_S1P and C_17_DH-S1P levels in the supernatants and cells were measured.

### Investigation of the distribution of C_17_S1P and C_17_DH-S1P between HDL and albumin

We prepared 1, 2, or 5 mg/ml of HDL obtained from healthy volunteers as described above and 10 mg/ml of albumin solution containing various concentrations of C_17_S1P, C_17_DH-S1P, or both. The samples were then incubated for 5 min at room temperature. After adjusting the density to 1.21 g/ml, we separated the solutions into HDL and albumin fractions once again using ultracentrifugation for 48 h. We measured the C_17_S1P or C_17_DH-S1P contents in the HDL fraction and the Lp-dep fraction.

For experiments investigating the binding of C_17_S1P or C_17_DH-S1P to HDL obtained from wild-type (WT) mice or apoM knockout (KO) mice, 1 µM C_17_S1P or C_17_DH-S1P bound to 10 mg/ml albumin was mixed with HDL collected from apoM KO mice or WT mice at 0.5 mg/ml. After incubation for 10 min, the samples were separated into HDL and Lp-dep fractions using ultracentrifugation.

### Animal experiments

The ApoM KO mice were produced as described in the previous report [17], and the blood samples were collected from 10-week-old male KO mice and WT mice, which were subjected to a 6-h fast. The plasma samples were separated into HDL and Lp-dep fractions, and the S1P and DH-S1P levels were measured.

For the experiments examining the overexpression of apoM, C57BL/6 mice were purchased from CLEA Japan (Tokyo, Japan). Ten-week-old male C57BL/6 mice were injected with an adenovirus coding apoM (Ad-apoM; generated as described in a previous report [12]) at a dose of 2.5 × 10^8^ pfu/g of body weight or a control blank adenovirus (Ad-null) at a dose of 2.5 × 10^8^ pfu/g of body weight via the tail vein. Five days after the injection of the adenoviruses, the mice were subjected to a 6-h fast and blood samples were subsequently collected.

All the animal experiments were conducted in accordance with the guidelines for Animal Care and were approved by the animal committee of The University of Tokyo.

### Cell experiments

HepG2 cells were cultured in DMEM supplemented with 10% fetal bovine serum and 1% penicillin/streptomycin in an incubator under 5% CO_2_. To obtain the conditioned medium of apoM-overexpressing HepG2 cells or control HepG2 cells, cells were infected with Ad-ApoM or Ad-null once they reached ∼80% confluency. After 2 days, the medium was replaced with serum-free medium; another 24 h later, the medium was collected and subjected to centrifugation and purification. The total volume (12 ml/dish) was then concentrated to ∼500 μl/dish using Amicon Ultra-15 (UFC901008; Millipore Co., Bedford, MA), as described previously [18,19]. We placed 5 μM C_17_S1P or C_17_DH-S1P bound to the conditioned medium of apoM-overexpressing HepG2 cells or control HepG2 cells on confluent HepG2 cells for 30 or 120 min. Then, the media were collected and the concentration of C_17_S1P or C_17_DH-S1P was measured.

### Western blot analysis

Western blotting was performed for each lipoprotein fraction separated from serum samples, using the standard method. The following antibodies were used: anti-human apoM serum (prepared as described in a previous article [12] and anti-apoA-I antibody (sc-30089; Santa Cruz Biotechnology).

### Measurement of S1P, DH-S1P C_17_-S1P, and C_17_DH-S1P

The contents of S1P, DH-S1P C_17_-S1P, and C_17_DH-S1P in the plasma, serum, and medium were determined using two-step lipid extraction followed by HPLC separation, as described previously [19]. Samples were sonicated in 3 ml of methanol/chloroform (2:1) with FTY720-phosphate (10006408; Cayman Chemical, Ann Arbor, MI) for 30 min Two milliliters of chloroform, 2.1 ml of 1 mM KCl, and 100 μl of 3 N NaOH were added to the sample and centrifuged at 2300 rpm for 15 min. After transferring 3.0 ml of the alkaline upper phase to new tubes, 4 ml of chloroform and 200 μl of HCl were added. The lower chloroform phases (3.5 ml) were collected and evaporated under nitrogen gas. The lipid extract was resolved in methanol, followed by HPLC separation with WakoPack Ultra C18-5 (232-02661; WAKO Pure Chemical Industries, Osaka, Japan). The flow rate was adjusted to 0.8 ml/min with 84% methanol in 0.05 mM K_2_HPO_4_/KH_2_PO_4_ (pH7.5). For the measurement of the S1P, DH-S1P, C_17_S1P and C_17_DH-S1P contents, we used FTY720-phosphate (10006408; Cayman Chemical, Ann Arbor, MI). S1P, DH-S1P, C_17_S1P, C_17_DH-S1P, and FTY720-phosphate were detected at Ex 350 nm/Em 450 nm.

### Validation of sphingolipid measurements using HPLC

A representative chromatograph is shown in Supplementary Figure S2A. We measured 1 μM of C_17_S1P and C_17_DH-S1P added to human serum samples using FTY720-phosphate as an internal standard. The average, standard deviation, and coefficient of variation are shown in Supplementary Table S1. Each coefficient of variation was less than 10%. To investigate the linearity of the present assay, different concentrations of a standard solution (0.016, 0.08, 0.4, 2.0, and 10 μM) were prepared and measured, and a linear relationship was confirmed up to a concentration of 10 μM (Supplementary Figure 2B–E).

### Statistical analysis

The results were expressed as the mean ± SD. Differences between two groups were evaluated using the Student *t*-test. *P* values less than 0.05 were deemed statistically significant.

## Results

### Distributions of S1P and DH-S1P among lipoprotein and lipoprotein-depleted fractions in human blood samples

**Figure 1:**
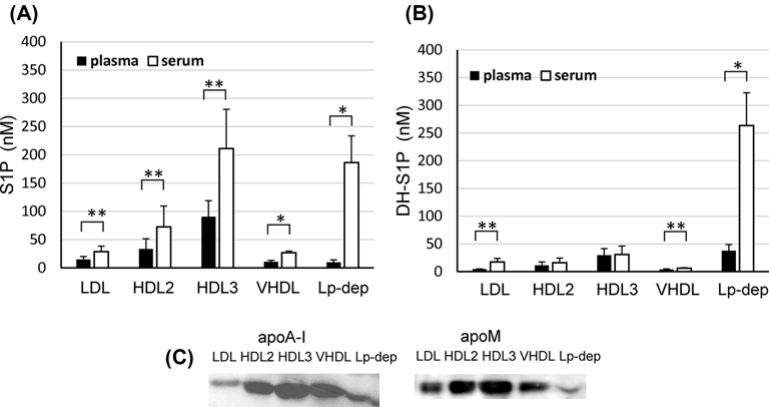
Distribution of S1P and DH-S1P in human blood samples Human lipoprotein fractions were prepared using ultracentrifugation and the levels of S1P (**A**) and DH-S1P (**B**) were measured (*n* = 5);*P<0.01, **P<0.05. The distribution of apoM and apoA-I was determined using a Western blot analysis (**C**).

First, we compared the distributions of S1P and DH-S1P among lipoprotein and Lp-dep fractions between human plasma and serum samples. We observed that S1P was mainly present in the HDL (HDL2 and HDL3) fraction, while DH-S1P was mainly present in the Lp-dep fraction in plasma. In serum samples, the S1P concentrations were 2.3 times higher in the HDL fraction and 18.8 times higher in the Lp-dep fraction than in the plasma sample, while the DH-S1P concentrations were 1.1 times higher in the HDL fraction and 7.0 times higher in the Lp-dep fraction than in the plasma sample (Figure 1A,B). These results suggest that platelet-derived S1P and DH-S1P (especially DH-S1P) might be preferentially distributed to the albumin fraction.

In both plasma and serum samples, the distribution patterns for HDL2 and HDL3 were not obviously different between S1P and DH-S1P. Since ApoM is a carrier of S1P, we compared the distributions of apoM in each lipoprotein fraction. ApoM was present mainly in the HDL2 and HDL3 fractions in all four of the examined subjects; representative results are shown in Figure 1C.

### Preference for HDL or albumin of C_17_S1P or C_17_DH-S1P released from platelets or erythrocytes

**Figure 2:**
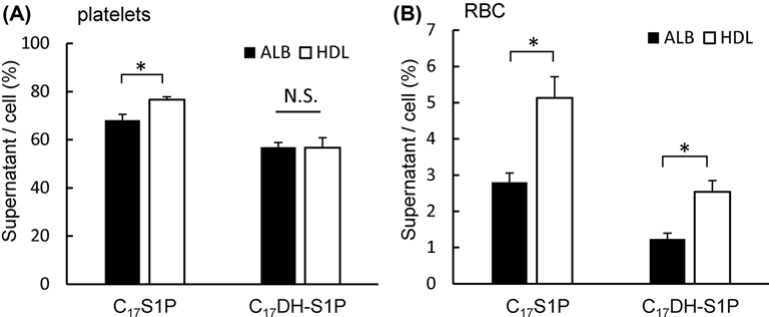
Distributions of C17S1P and C17DH-S1P secreted from platelets or red blood cells between albumin and HDL Washed platelets (**A**) or red blood cells (**B**) were incubated with C_17_Sph or C_17_DH-Sph and albumin or HDL. After washing, cellular and supernatant samples were separated into HDL and lipoprotein-depleted fractions by ultracentrifugation, and the C_17_S1P and C_17_DH-S1P levels were measured. The ratios of supernatant C_17_S1P or C_17_DH-S1P to the cellular contents are shown; *P<0.01.

Since the distributions of S1P and DH-S1P among the HDL and Lp-dep fractions differed according to the type of blood sample (serum or plasma), we next investigated the preference for HDL or albumin of C_17_S1P and C_17_DH-S1P produced from activated platelets or erythrocytes. As shown in Figure 2A, activated platelets produced and released both C_17_S1P and C_17_DH-S1P; C_17_S1P released from platelets was distributed to HDL more than albumin, whereas C_17_DH-S1P released from platelets was equally distributed to HDL and albumin. As for the erythrocytes, HDL accepted both C_17_S1P and C_17_DH-S1P transported from erythrocytes more than albumin (Figure 2B). These results suggest that HDL might possess a lower affinity to platelet-derived DH-S1P than to S1P.

### C_17_S1P and C_17_DH-S1P were distributed to both HDL and albumin fractions but preferred to be bound to HDL

**Figure 3:**
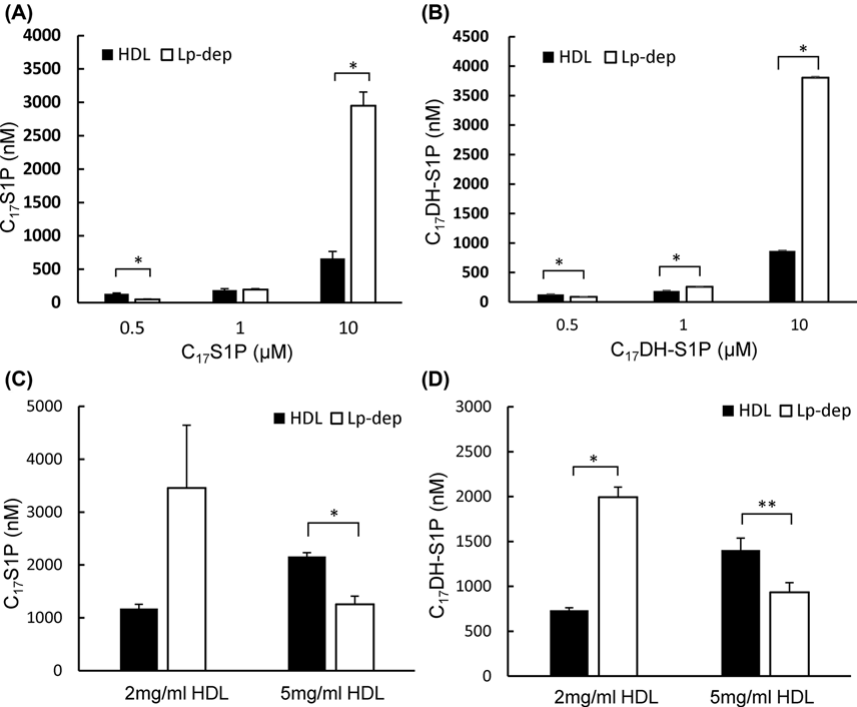
Effects of the concentrations of C17S1P and C17DH-S1P or HDL on their distributions in HDL and albumin fractions (**A** and** B**) C_17_S1P or C_17_DH-S1P at concentrations of 0.5, 1.0, or 10 μM was incubated with 1 mg/ml of HDL and 10 mg/ml of albumin, and the HDL or albumin fractions were separated. The C_17_S1P and C_17_DH-S1P levels were then measured in each fraction. (**C** and **D**) C_17_S1P or C_17_DH-S1P at a concentration of 1 µM was mixed with 2 or 5 mg/ml of HDL and 10 mg/ml of albumin, and the HDL and albumin fractions were separated. The C_17_S1P and C_17_DH-S1P levels were then measured and the distributions of these sphingolipids were compared; *P<0.01 and **P<0.05.

To compare the capacity of HDL and albumin to receive S1P or DH-S1P, we next investigated how C_17_S1P or C_17_DH-S1P were distributed between the HDL and Lp-dep fractions using various concentration of C_17_S1P and C_17_DH-S1P. As shown in Figure 3A,B, both C_17_S1P and C_17_DH-S1P showed a preference for HDL (1 mg/ml) at a concentration of 0.5 μM; at 10 μM, however, larger amounts of C_17_S1P and C_17_DH-S1P were present in the Lp-dep fraction. When various concentrations of HDL were used as an acceptor, both C_17_S1P and C_17_DH-S1P were shifted from the lipoprotein-depleted fraction to the HDL fraction, depending on the concentration of HDL (Figure 3C,D). These results suggest that HDL can accept larger amounts of S1P and DH-S1P than albumin.

### HDL preferentially bonded to C_17_S1P rather than C_17_DH-S1P

**Figure 4:**
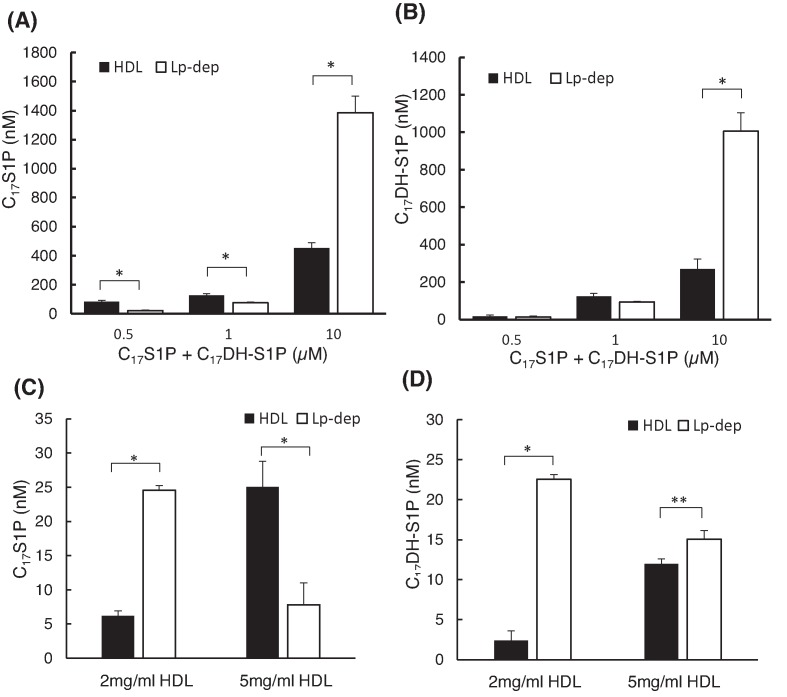
Distributions of co-existing C17S1P and C17DH-S1P in HDL and albumin fractions (**A** and **B**) Both C_17_S1P and C_17_DH-S1P were added to 1 mg/ml of HDL and 10 mg/ml of albumin, and the HDL and albumin fractions were separated. The levels of C_17_S1P and C_17_DH-S1P were then measured. (**C** and **D**) Both C_17_S1P and C_17_DH-S1P were added to 2 or 5 mg/ml of HDL and 10 mg/ml of albumin, and the HDL and albumin fractions were separated. The C_17_S1P and C_17_DH-S1P levels were then measured and the distributions of these sphingolipids were compared; *P<0.01, **P<0.05.

To determine whether HDL prefers to accept S1P or DH-S1P, we investigated how C_17_S1P and C_17_DH-S1P were distributed between HDL and albumin in a situation where both C_17_S1P and C_17_DH-S1P were present. Although the patterns of their distributions were almost the same (Figure 4A,B), when we used a higher concentration of HDL (5 mg/dl), C_17_S1P was preferentially distributed to the HDL fraction, while C_17_DH-S1P was distributed to the albumin fraction (Figure 4C,D), suggesting that HDL might prefer S1P over DH-S1P.

### S1P, but not DH-S1P, was hardly detected on HDL in apoM knockout mice

**Figure 5:**
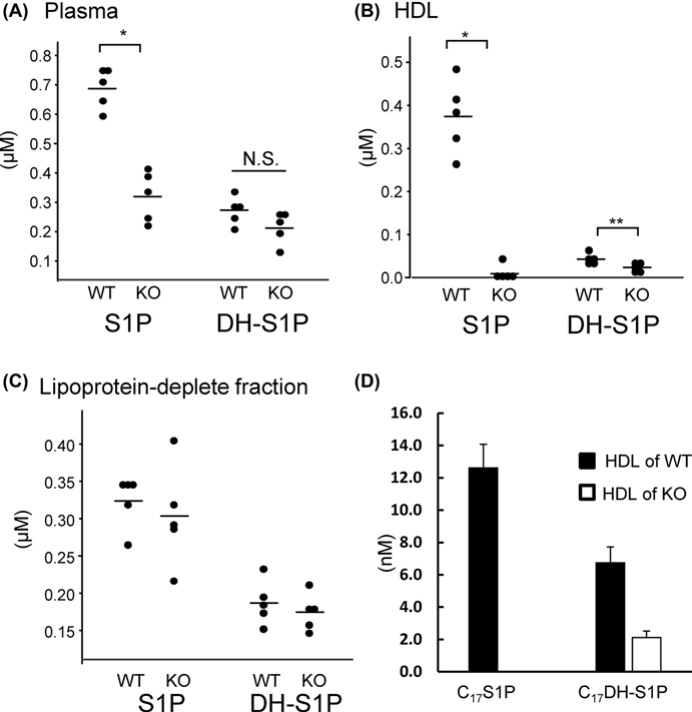
S1P and DH-S1P contents in plasma, HDL, and lipoprotein-depleted fractions obtained from apoM knockout mice (**A**–**C**) Plasma samples were collected from 10-week-old wild-type (WT) mice and apoM knockout (KO) mice after 4 h of fasting. The plasma samples were then separated into HDL and lipoprotein-depleted fractions. The S1P and DH-S1P levels were measured in the total plasma (A), HDL (B), and lipoprotein-depleted fractions (C). (**D**) C_17_S1P or C_17_DHS1P (1 µM) bound to albumin was mixed with HDL collected from apoM KO mice or WT mice at a concentration of 0.5 mg/ml. After 10 min of incubation, the samples were separated into HDL and lipoprotein-depleted fractions using ultracentrifugation. Then, the C_17_S1P and C_17_DHS1P levels in each fraction were measured (*n*=5); *P<0.01, **P<0.05.

Since we observed differences in the distributions of S1P and DH-S1P between HDL and albumin, we next investigated the affinities of S1P and DH-S1P to apoM**-**deficient HDL. When we measured the S1P and DH-S1P levels in plasma, HDL, and the Lp-dep fraction in WT mice and apoM KO mice, we found that the plasma S1P levels were lower in apoM KO mice, while the plasma DH-S1P levels were not statistically different (Figure 5A). In the HDL fraction, S1P was hardly detectable on HDL from KO mice; DH-S1P was detectable, but the DH-S1P level was lower for HDL from KO mice (Figure 5B). Regarding the Lp-dep fraction, no significant differences were observed between the WT mice and the apoM KO mice (Figure 5C). These results suggested that apoM might bind S1P, but not DH-S1P. Actually, when we incubated 10 nM C_17_S1P or C_17_DH-S1P with albumin and HDL separated from apoM KO mice or WT mice, we observed that C_17_S1P was distributed only to HDL obtained from WT mice, while C_17_DH-S1P was distributed to HDL from both WT mice and apoM KO mice (Figure 5D). These results suggest that S1P might bind to HDL in a specific manner via apoM, while DH-S1P might bind in a non-specific manner independent of apoM.

### S1P and DH-S1P were increased in HDL fractions from apoM-overexpressing mice

**Figure 6:**
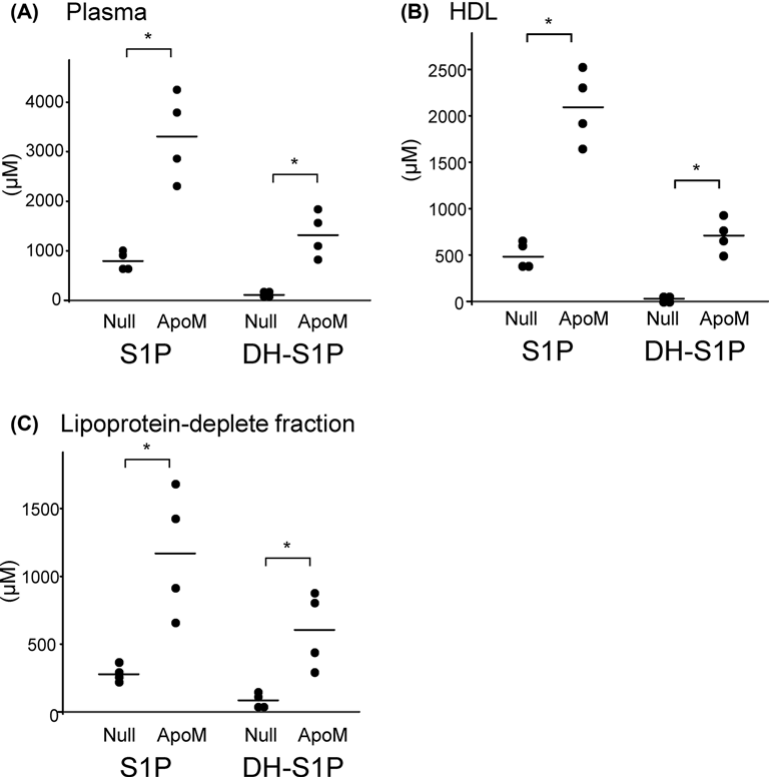
S1P and DH-S1P contents in plasma, HDL, and lipoprotein-depleted fractions obtained from apoM-overexpressing mice Ten-week-old male C57BL/6 mice were injected with adenovirus coding apoM (ApoM) or control blank adenovirus (Null). Five days after the injection of the adenoviruses, the mice were subjected to a 6-h fast, and plasma samples were collected and subsequently separated into HDL and lipoprotein-depleted fractions. The S1P and DH-S1P levels were measured in the total plasma (**A**), HDL (**B**), and lipoprotein-depleted fractions (**C**); *P<0.01

We also investigated the distributions of S1P and DH-S1P between HDL and Lp-dep fractions in apoM-overexpressing mice. As shown in Figure 6, the S1P and DH-S1P levels in the total plasma, HDL, and Lp-dep fractions were elevated in apoM-overexpressing mice. These results agree with the finding that excessive amounts of S1P and DH-S1P might be distributed not only to HDL, but also to albumin.

### ApoM retarded the degradation of C_17_S1P, but not C_17_DH-S1P

**Figure 7:**
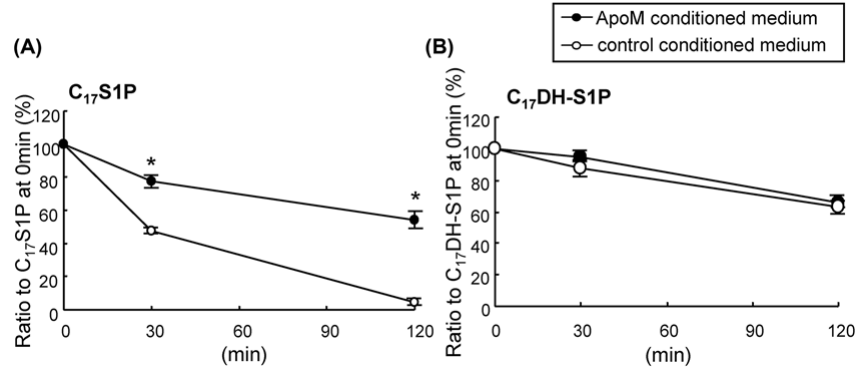
Effects of apoM on the degradation of C17S1P or C17DHS1P on HepG2 cells C_17_S1P or C_17_DH-S1P (5 μM) bound to the conditioned medium of apoM-overexpressing HepG2 cells or control HepG2 cells was incubated on confluent HepG2 cells for 30 or 120 min (*n*=4). The media were collected, and the concentrations of C_17_S1P or C_17_DH-S1P were measured. The data were expressed as the percentage of C_17_S1P or C_17_DH-S1P at 30 or 120 min to those at 0 min; *P<0.01

Finally, we investigated the physiological significance of the different HDL-binding mechanisms between S1P and DH-S1P. Since apoM retards the degradation of S1P on cells, we investigated whether apoM might influence the degradation of DH-S1P. When the conditioned medium of apoM-overexpressing HepG2 cells was used as a vehicle for C_17_S1P or C_17_DH-S1P, we observed that the degradation of C_17_S1P on other confluent HepG2 cells was retarded, while that of C_17_DH-S1P was not modulated (Figure 7). These results suggest that the protective effects of apoM on the degradation of S1P were not observed for DH-S1P.

## Discussion

S1P has multiple physiological functions, such as antiapoptosis, cell proliferation, vasorelaxation, and the maintenance of vascular permeability. DH-S1P, an analog of S1P, has been proposed to possess similar functions. However, the functions and kinetics of DH-S1P have not been adequately investigated. In the present study, we investigated the difference between S1P and DH-S1P in the kinetics of these sphingolipids, especially regarding their associations with apoM/HDL.

First, we compared the distributions of S1P and DH-S1P among lipoproteins between human plasma and serum samples and observed that increased amounts of DH-S1P in serum samples were distributed to the Lp-dep fraction to a greater degree than to the HDL fraction, compared with increased S1P levels (Figure 1). These results suggest that platelet-derived DH-S1P might be preferentially distributed to the albumin fraction, since plasma S1P and DH-S1P mainly reflect molecules transported from erythrocytes [6], while serum S1P and DH-S1P are also derived from platelets [20]. Actually, although S1P secreted from activated platelets was distributed to HDL to a greater degree than albumin, DH-S1P was similarly distributed to albumin and HDL (Figure 2A). Although the mechanisms responsible for the secretion of S1P and DH-S1P from platelets have not been fully elucidated, S1P in cell membrane and granular pools is thought to be secreted when the platelets are activated [21]. Therefore, some difference might exist between S1P and DH-S1P associated with platelets such as the distributions within the cell membrane and granules in platelets. Regarding S1P or DH-S1P transported from erythrocytes, we observed that HDL served as a higher acceptor of S1P and DH-S1P than albumin (Figure 2B). This result agreed with the findings of previous papers demonstrating that HDL can accept S1P secreted from erythrocytes to a greater extent than albumin [22–24]. Regarding DH-S1P, however, this was the first study to demonstrate that HDL can accept DH-S1P secreted from erythrocytes to a greater extent than albumin, which seems reasonable since both DH-S1P and S1P are carried on HDL (Figures 1 and 6).

Next, we demonstrated preferences for S1P and DH-S1P to be bound to HDL rather than albumin (Figure 3), while HDL preferentially accepts S1P over DH-S1P (Figure 4). Regarding the affinity of HDL and albumin to S1P and DH-S1P, considering the molecular weight of HDL (185–386 kDa) [25] and albumin (66 kDa) and the results of Figure 3C,D, we estimate that S1P and DH-S1P prefer HDL over albumin by 4.7–9.1 times and by 3.5–7.3 times, when HDL at 2 mg/ml was used, and by 11.2–23.4 times and by 8.4–17.5 times, when HDL at 5 mg/ml used. Moreover, when we estimated from the results of Figure 4C,D, since C_17_S1P bound to HDL twice more than C_17_DH-S1P, the affinity of HDL for S1P might be twice higher than DH-S1P.

Although the affinity of DH-S1P to HDL or albumin has not been investigated, an elegant study elucidated that the binding affinity of albumin to S1P was lower than that of apoM/HDL [26], which agrees with the results from the present study. Moreover, the present study suggests that when S1P and DH-S1P exist in excess of the capacity of HDL, they might be distributed to albumin (Figure 3A,B). These results partially explain the reason for the relatively weak correlation between S1P and apoM in human plasma [27]. Considering the different physiological properties between S1P bound to apoM and to albumin, as described below, these results also suggest that the excessive production of S1P might have harmful effects, since S1P that cannot bind to HDL might instead bind to albumin.

At last, we investigated the affinity of DH-S1P to HDL/apoM and found that DH-S1P might bind to HDL in a non-specific manner, since DH-S1P, but not S1P, was detected in HDL separated from apoM KO mice and C_17_DH-S1P, but not C_17_S1P, was bound to apoM-deficient HDL (Figure 5). Interestingly, however, a smaller amount of DH-S1P was detected in apoM-deficient HDL than in HDL obtained from WT mice. Furthermore, DH-S1P in plasma or HDL was also increased by the overexpression of apoM (Figure 6), similar to the results of previous studies [12,28]. These results suggest that apoM might affect the metabolism of DH-S1P in a manner different from its effect on the metabolism of S1P, which was the main unresolved issue of the present study. Actually apoM retarded the degradation of C_17_S1P but did not affect the kinetics of C_17_DH-S1P (Figure 7). In a previous study demonstrating that both intracellular S1P and DH-S1P levels were increased in apoM-overexpressing mice, as well as plasma S1P and DH-S1P levels [12], we speculated that apoM might bind to intracellular S1P and DH-S1P and prevent them from degradation, as was observed outside the cells. However, the results that DH-S1P might not be bound to apoM, while DH-S1P levels increased in apoM-overexpressing mice (Figure 6) and decreased in apoM knockout mice (Figure 5) suggest another mechanism in which apoM directly or indirectly affects the intracellular S1P and DH-S1P levels. In addition to the roles of the carrier and modulator of S1P, apoM reportedly has important roles in lipid homeostasis; for example, apoM modulates the metabolism and distribution of cholesterol, triglycerides, and phospholipids in the plasma, livers, and adipose tissues [12,28,29]. Further studies might elucidate novel roles of apoM in lipid metabolism including DH-S1P.

Although the physiological significance of the apoM-independent binding of DH-S1P to HDL was not determined in the present study, this finding might be important for identifying the clinical significance of DH-S1P, since emerging reports have demonstrated possible differences in physiological properties between albumin-bound S1P and apoM/HDL-bound S1P: S1P bound to apoM had a greater effect on the maintenance of the endothelial barrier [30] and on the secretion of insulin from pancreatic β cells [31], while S1P carried on apoM had a suppressive effect on the inflammation of endothelial cells [32], the proliferation of lymphocytes [33], and the induction of PAI-1 [34]. Since DH-S1P might not be bound to apoM, the biological properties of DH-S1P might not be affected by its carrier. Therefore, DH-S1P might be a biomarker for several diseases independent from S1P. Further studies are necessary to elucidate the clinical significance of DH-S1P.

In summary, although DH-S1P binds preferentially to HDL, its affinity might be lower than that of S1P, and DH-S1P binds to HDL in an apoM-independent manner. Thus, DH-S1P is not a mere analog of S1P, but might possess unique clinical significance.

## Supporting information

**Supplemental Figure 1.** The structures of S1P and DH-S1P.

**Supplemental Figure 2.** Linearity of sphingolipid measurements. (A) Representative chromatograms obtained using an HPLC method to measure sphingolipid levels. The linearity of (B) S1P, (C) DH-S1P, (D) C_17_S1P, and (E) C_17_DH-S1P was observed using 0.016 μM, 0.08 μM, 0.4 μM, 2.0 μM, and 10 μM standard samples (n = 4).

**Supplemental Table S1:** 

## Abbreviations

Ad-apoM: adenovirus coding apoM
Ad-null: control blank adenovirus
ApoM: apolipoprotein M
DH-S1P: dihydro-sphingosine 1-phosphate
DH-Sph: dihydro-sphingosine
KO: knockout
Lp-dep: lipoprotein**-**depleted
S1P: sphingosine 1-phosphate
Sph: sphingosine
WT: wild-type
