# Supplementary material for: Dihydro-sphingosine 1-phosphate interacts with carrier proteins in a manner distinct from that of sphingosine 1-phosphate
Source: Biosci Rep. 2018 Oct 31;38(5):BSR20181288. doi: 10.1042/BSR20181288 (PMC6209608; doi:10.1042/BSR20181288)
Supplement: Supplementary file 1 [file bsr20181288_Supp1.pdf]

Supplementary Table 1.

	S1P (nM)	DH-S1P (nM)	C_{17}S1P (nM)	C_{17}DH-S1P (nM)
Average	1027.6	411.8	1041.1	1023.9
SD	98.4	38.6	99.5	94.0
CV (%)	9.6	9.4	9.6	9.2

Validation of sphingolipid measurements using HPLC.

Supplementary Figure 1

A **S1P**

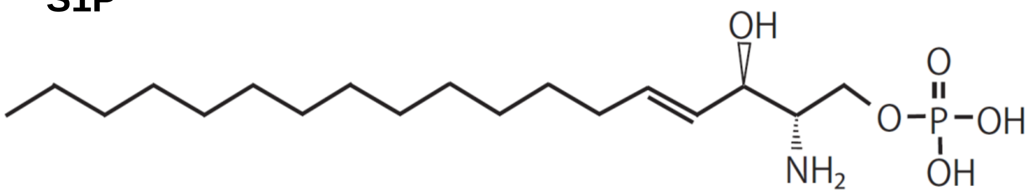

B **DH-S1P**

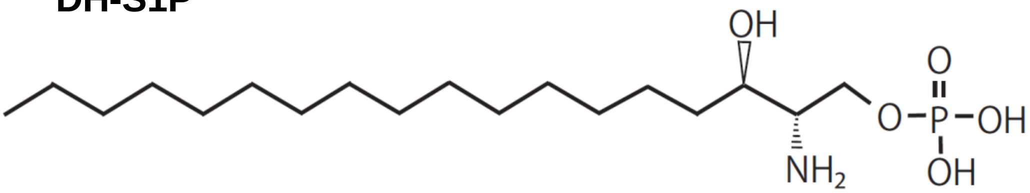

Supplemental Figure 1. The structures of S1P and DH-S1P.

Supplementary Figure 2

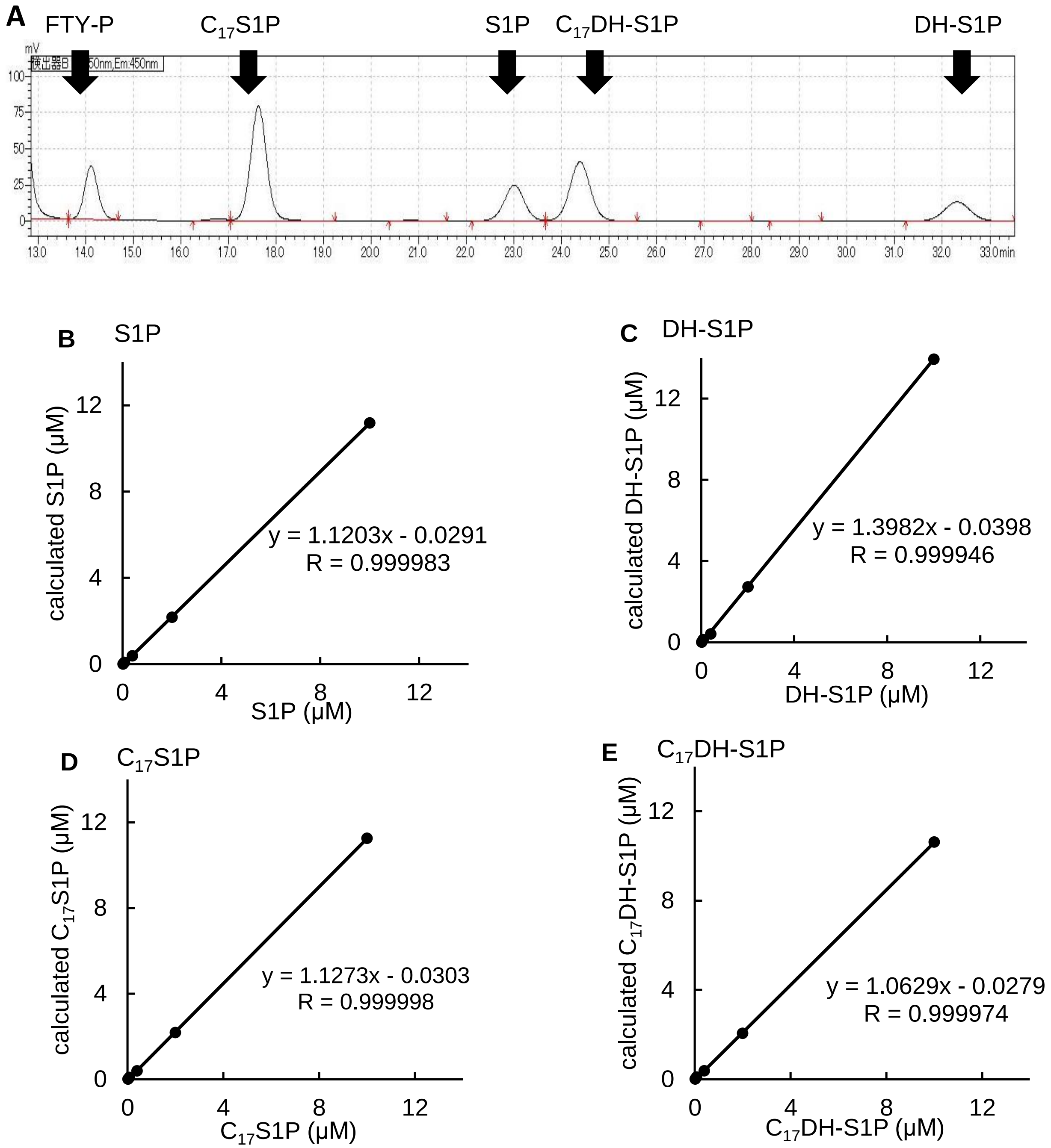

Supplementary Figure 2. Linearity of sphingolipid measurements. (A) Representative chromatograms obtained using an HPLC method to measure sphingolipid levels. The linearity of (B) S1P, (C) DH-S1P, (D) C_{17}S1P, and (E) C_{17}DH-S1P was observed using 0.016 μM, 0.08 μM, 0.4 μM, 2.0 μM, and 10 μM standard samples (n = 4).
